# Supplementary material for: Associations of Obstructive Sleep Apnea, Obestatin, Leptin, and Ghrelin with Gastroesophageal Reflux
Source: J Clin Med. 2021 Nov 7;10(21):5195. doi: 10.3390/jcm10215195 (PMC8584398; doi:10.3390/jcm10215195)
Supplement: Supplementary file 1 [file jcm-10-05195-s001.zip › jcm-1410009-supplementary.pdf]

KWESTIONARIUSZ OBJAWÓW REFLUKSOWYCH

DATA …................. NAZWISKO i IMIĘ …..

Telefon kontaktowy: …..

Zgaga- uczucie pieczenia lub palenia za mostkiem które promieniuje do szyi i gardła, któremu towarzyszy cofanie się kwaśnej treści do jamy ustnej.

Poniższa skala w postaci strzałki ujmuje dokuczliwość objawów zgagi od 0- brak objawów; do 100- skrajna dokuczliwość (objawy są bardzo częste i niezwykle silne).

Proszę ocenić **dokuczliwość zgagi u Pana(-i) w okresie ostatniego miesiąca,** zakreślając na linii kreskę w wybranym miejscu między 0 a 100.

W ostatnim miesiącu objawy zgagi

w ogóle nie występowały

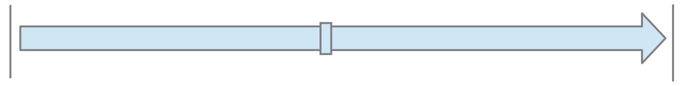

objawy były skrajnie dokuczliwe

0 50 100

Na poniższym rysunku proszę zaznaczyć obszar,
w którym zazwyczaj odczuwa Pan(i) pieczenie lub palenie.

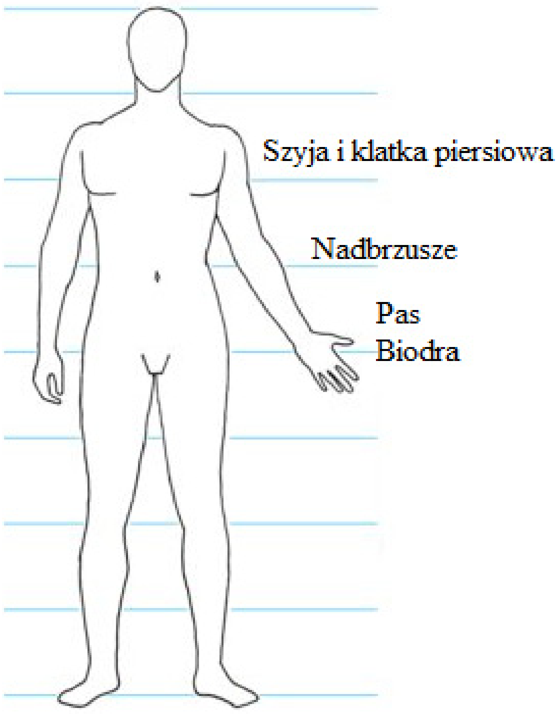

OBJAWY W OKRESIE OSTATNIEGO MIESIĄCA

Proszę zakreślić kółkiem wybraną odpowiedź

1/ Czy uczucie pieczenia/palenia w klatce piersiowej pojawiało się **po posiłkach**

JAK CZĘSTO?	0	1	2	3	4
	**nie występowało**	**sporadycznie** 1-2 razy w ciągu miesiąca	**rzadko** raz w tygodniu lub rzadziej	**często** kilka razy w tygodniu	**bardzo często** prawie po każdym posiłku
Z JAKIM NASILENIEM?		1	2	3	4
		**łagodne** nie zwracające uwagi	**umiarkowane** zwracało uwagę	**silne** przeszkadzające	**bardzo silne** trudne do zniesienia

2/ Czy uczucie pieczenia/palenia pojawiało się **w pozycji leżącej**?

JAK CZĘSTO?	0	1	2	3	4
	**nie występowało**	**sporadycznie** 1-2 razy w ciągu miesiąca	**rzadko** raz w tygodniu lub rzadziej	**często** kilka razy w tygodniu	**bardzo często** prawie zawsze
Z JAKIM NASILENIEM?		1	2	3	4
		**łagodne** nie zwracające uwagi	**umiarkowane** zwracało uwagę	**silne** przeszkadzające	**bardzo silne** trudne do zniesienia

3/ Czy uczucie pieczenia/palenia pojawiało się **przy schylaniu**?

JAK CZĘSTO?	0	1	2	3	4
	**nie występowało**	**sporadycznie** 1-2 razy w ciągu miesiąca	**rzadko** raz w tygodniu lub rzadziej	**często** kilka razy w tygodniu	**bardzo często** prawie przy każdej próbie schylania się
Z JAKIM NASILENIEM?		1	2	3	4
		**łagodne** nie zwracające uwagi	**umiarkowane** zwracało uwagę	**silne** przeszkadzające	**bardzo silne** trudne do zniesienia

4/ Czy występowały **odbijania** lub uczucie **cofania się pokarmu z żołądka** z pozostawianiem kwaśnego lub gorzkiego smaku w jamie ustnej?

JAK CZĘSTO?	0	1	2	3	4
	**nie występowało**	**sporadycznie** 1-2 razy w ciągu miesiąca	**rzadko** raz w tygodniu lub rzadziej	**często** kilka razy w tygodniu	**bardzo często** codziennie lub prawie codziennie
Z JAKIM NASILENIEM?		1	2	3	4
		**łagodne** nie zwracające uwagi	**umiarkowane** zwracało uwagę	**silne** przeszkadzające	**bardzo silne** trudne do zniesienia

5/ Czy miał (-a) Pan (-i) uczucie **zatrzymywania się połykanego pokarmu** w gardle lub w przełyku?

	0	1	2	3	4
JAK CZĘSTO?	**nie występowało**	**sporadycznie**<br>1-2 razy w ciągu miesiąca	**rzadko**<br>raz w tygodniu lub rzadziej	**często**<br>kilka razy w tygodniu	**bardzo często**<br>przy większości prób połykania

	1	2	3	4
Z JAKIM NASILENIEM?	**łagodne**<br>nie zwracające uwagi	**umiarkowane**<br>zwracało uwagę	**silne**<br>przeszkadzające	**bardzo silne**<br>trudne do zniesienia

6/ Czy uczucie pieczenia/palenia w klatce piersiowej **utrudniało zaśnięcie lub wybudzało** Pana/Panią w nocy **ze snu**?

	0	1	2	3	4
JAK CZĘSTO?	**nie występowało**	**sporadycznie**<br>1-2 razy w ciągu miesiąca	**rzadko**<br>raz w tygodniu lub rzadziej	**często**<br>kilka razy w tygodniu	**bardzo często**<br>co noc lub prawie co noc

7/ Czy zdarzało się, że miał Pan (-i) uczucie kwaśnego lub gorzkiego smaku w ustach **po nocnym wypoczynku**?

	0	1	2	3	4
JAK CZĘSTO?	**nie występowało**	**sporadycznie**<br>1-2 razy w ciągu miesiąca	**rzadko**<br>raz w tygodniu lub rzadziej	**często**<br>kilka razy w tygodniu	**bardzo często**<br>codziennie lub prawie codziennie

8/ Czy zdarzało się, że **odbijania** lub uczucie **cofania się pokarmu z żołądka do** z pozostawianiem kwaśnego lub gorzkiego smaku w jamie ustnej **utrudniało zaśnięcie lub wybudzało** Pana/Panią w nocy **ze snu?**

	0	1	2	3	4
JAK CZĘSTO?	**nie występowało**	**sporadycznie**<br>1-2 razy w ciągu miesiąca	**rzadko**<br>raz w tygodniu lub rzadziej	**często**<br>kilka razy w tygodniu	**bardzo często**<br>co noc lub prawie co noc

9/ Jak często w okresie ostatniego miesiąca pojawiały się u Pana/Pani:

	❶ 0 **wcale**	1 **sporadycznie** 1-2 razy w ciągu miesiąca	2 **rzadko** raz w tygodniu lub rzadziej	3 **często** kilka razy w tygodniu	4 **bardzo często** codziennie lub prawie codziennie
Bóle w klatce piersiowej					
Uczucie ropierania w klatce piersiowej w trakcie jedzenia					
Ból za mostkiem przy połykaniu					
Uczucie pełności w nadbrzuszu					
Nudności					
Wymioty					
Uczucie dławienia w gardle					
Krztuszenie się w trakcie jedzenia					
Suchość w ustach					
Chrypka					
Przewlekły kaszel					
Uczucie duszności					

OBJAWY KLINICZNE GERD:

Suma punktów:	dzienne (ptk.1-5):	....
	nocne (ptk.6-8):	....
	inne (ptk.9):	....

BADANIE PODMIOTOWE

Wzrost:____________

Waga:_____________

Obwód szyi:________

Obwód pasa:_______

Miejsce wkłucia: ....

23^{00} : ....

1^{00} : ....

3^{00} : ....

5^{00} : ....

7^{00} : ....

Przyjmowane leki:____

PPI/H_2-B (w dawce)____

Leki nasilające bezdech:____

Istotne dla OSA lub GERD i greliny, przebyte operacje (twarzoczaszki, operacje na przewodzie pokarmowym np. resekcja żołądka):____________________________________

____

____

____

____

Proszę o zaznaczenie na poniższej tabeli prawdopodobieństwa zaśnięcia we wskazanych sytuacjach w ciagu ostatnich 6 tygodni.

0 – zerowe prawdopodobieństwo zaśnięcia	1 – małe prawdopodobieństwo zaśnięcia
2 – średnie prawdopodobieństwo zaśnięcia	3 – duże prawdopodobieństwo zaśnięcia

Sytuacja	Proszę zaznaczyć właściwą odpowiedź
Siedzenie i czytanie	0 1 2 3
Oglądanie telewizji	0 1 2 3
Bierne siedzenie w miejscach publicznych (np. w teatrze, na zebraniu)	0 1 2 3
Jako pasażer w samochodzie, jadąc przez godzinę bez odpoczynku	0 1 2 3
Leżenie i odpoczywanie po południu, jeśli okoliczności na to pozwalają	0 1 2 3
W czasie rozmowy, siedząc	0 1 2 3
Spokojne siedzenie po obiedzie bez alkoholu	0 1 2 3
W samochodzie, podczas kilkuminutowego postoju w korku lub na czerwonym świetle	0 1 2 3

Kwestionariusz berliński.

Proszę zaznaczyć odpowiedź przy każdym pytaniu.

KATEGORIA 1

1. Czy chrapiesz?

☐ a. Tak

☐ b. Nie

☐ c. Nie wiem

Jeżeli chrapiesz to:

2. Czy twoje chrapanie jest:

☐ a. Nieco głośniejsze niż oddychanie

☐ b. Tak głośne jak mowa

☐ c. Głośniejsze niż mowa

☐ d. Bardzo głośne – może być słyszalne w sąsiednich pomieszczeniach

3. Jak często chrapiesz?

☐ a. Prawie zawsze

☐ b. 3-4 razy w tygodniu

☐ c. 1-2 razy w tygodniu

☐ d. 1-2 razy w miesiącu

☐ e. Nigdy lub prawie nigdy

4. Czy twoje chrapanie przeszkadza innym osobom?

☐ a. Tak

☐ b. Nie

☐ c. Nie wiem

5. Czy kiedykolwiek ktoś zauważył, że przestajesz oddychać podczas snu?

☐ a. Prawie zawsze

☐ b. 3-4 razy w tygodniu

☐ c. 1-2 razy w tygodniu

☐ d. 1-2 razy w miesiącu

☐ e. Nigdy lub prawie nigdy

KATEGORIA 2

6. Jak często czujesz się zmęczony po przebudzeniu?

☐ a. Prawie zawsze

☐ b. 3-4 razy w tygodniu

☐ c. 1-2 razy w tygodniu

☐ d. 1-2 razy w miesiącu

☐ e. Nigdy lub prawie nigdy

7. Jak często w ciągu dnia czujesz się zmęcz[illegible] lub jesteś w gorszej formie?

☐ a. Prawie zawsze

☐ b. 3-4 razy w tygodniu

☐ c. 1-2 razy w tygodniu

☐ d. 1-2 razy w miesiącu

☐ e. Nigdy lub prawie nigdy

8. Czy kiedykolwiek przysypiałeś lub zasnąłeś prowadząc pojazd mechaniczny?

☐ a. Tak

☐ b. Nie

Jeżeli Tak to:

9. Jak często to się zdarza?

☐ a. Prawie zawsze

☐ b. 3-4 razy w tygodniu

☐ c. 1-2 razy w tygodniu

☐ d. 1-2 razy w miesiącu

☐ e. Nigdy lub prawie nigdy

KATEGORIA 3

10. Czy masz nadciśnienie tętnicze?

☐ Tak

☐ Nie

☐ Nie wiem

Osobna strona (w załączeniu): polisomno/poligrafia; wyniki badań lab. i oznaczeń laboratoryjnych
